# Construction of 3D bioprinted colorectal cancer models and evaluation of their efficacy and drug sensitivity

**DOI:** 10.1063/5.0320497

**Published:** 2026-08-10

**Authors:** Feng Tong, Ruijin Nie, Zhenyu Wu, Longfei Zhang, Xia Zhang, Xiaoyu Duan, Qi Zhang, Wen Tang

**Affiliations:** 1 Department of General Surgery, Lanxi People's Hospital, Lanxi 321100, Zhejiang, China; 2 Department of Pathology, Lanxi People's Hospital, Lanxi 321100, Zhejiang, China; 3 Department of Endocrinology, Lanxi People's Hospital, Lanxi 321100, Zhejiang, China; 4 Saibo (Shanghai) Intelligent Technology Co., Ltd., Shanghai, China

## Abstract

Currently, preclinical research on colorectal cancer (CRC) lacks effective tumor models. In recent years, 3D bioprinted models have shown great potential in tumor model construction. This study used 3D bioprinting technology to establish a CRC extracellular matrix (ECM) model. Gradient concentrations of laminin/entactin complex combined with photocurable gelatin methacrylate (GelMA) hydrogel were used to prepare 3D bioprinting bioinks with biomimetic ECM properties. Biocompatibility was assessed by calcein-AM and propidium iodide staining. Proliferation was evaluated using the CellTiter-Glo^®^ Luminescent Cell Viability Assay Kit in SW620 cells and primary CRC cells. A “sandwich structure” model with inner and outer layers of GelMA or GelMA + LE2 was constructed to observe cell invasion. Mechanical properties of GelMA + LE2 were characterized. Total RNA was extracted from cells in 3D bioprinted models constructed with GelMA and GelMA + LE2 for transcriptome sequencing. Finally, chemotherapeutic drug tests were performed on the 3D bioprinted models. A 3D bioprinted CRC organoid model based on GelMA + LE2 was successfully established. This model exhibited good biocompatibility and the ability to promote tumor cell proliferation and invasion. These characteristics were independent of the model's mechanical properties. Instead, they originated from LE2 activating the ErbB/Wnt signaling pathway and gap junction-mediated intercellular communication. Additionally, a significant correlation was observed between the clinical treatment outcomes of CRC patients and the drug test results of the 3D bioprinted models. The 3D bioprinted CRC organoid constructed in this study provides a biomimetic and high-throughput research platform for precision medicine.

## I. INTRODUCTION

Colorectal cancer (CRC) has become the third most common malignant tumor worldwide and the second leading cause of cancer-related deaths.^1^ In China, the incidence and mortality rates of CRC have shown a significant increasing trend, ranking second and fourth among all malignant tumors, respectively.^2^ Approximately 20% of CRC patients are diagnosed at stage IV, which is incurable by surgical resection. They require systemic treatment,^3^ and their 5-year relative survival rate is as low as 14%.^4^ Therefore, formulating personalized treatment plans for different stage IV CRC patients is particularly important.^5^ However, the high heterogeneity and dynamic development of CRC as well as the complex microenvironmental components and highly immunosuppressive microenvironment within tumors pose great challenges to treatment.^6,7^ To address these challenges, developing *in vitro* and *in vivo* models for CRC diagnosis and treatment and conducting accurate efficacy evaluation are of great significance.

The currently widely used preclinical research models include 2D cell models and patient-derived xenograft (PDX) models. However, both have limitations in simulating the CRC tumor microenvironment. Due to the single type of cultured cells and lack of extracellular matrix (ECM), 2D cell models usually cannot effectively maintain tumor cell heterogeneity or reproduce the diversity and complexity of tumor tissues.^8^ As an animal model closer to the real tumor microenvironment, PDX has been proven useful in predicting tumor drug sensitivity or resistance to improve guidance for patient treatment.^9^ Nevertheless, the defects of PDX models cannot be ignored, such as individual differences, long culture cycles, low success rates, and species differences from humans. In recent years, patient-derived organoid (PDTO) models have been extensively studied.^10^ They can not only makeup for the inability of traditional 2D cell models to maintain cell heterogeneity but also have a shorter culture cycle and better cost-effectiveness than PDX models. Even so, PDTO still faces problems such as the lack of standardized methods and large differences between samples.^11^ Crucially, none of these models can concurrently achieve high repeatability, controlled extracellular matrix (ECM) composition, quick culture cycles, and realistic simulation of tumor heterogeneity, despite the fact that each model has separately improved CRC research. A preclinical model that combines standardized and clinically applicable manufacturing techniques with biomimetic tumor microenvironments is therefore still desperately needed.

In addition to these popular tumor models, 3D bioprinted models have emerged as an advanced biofabrication technology in emerging studies. They can combine multiple cells and extracellular matrix according to preset structures to construct complex *in vitro* bioactive tissue models.^12^ This technology has made significant progress in tumor research and tissue repair. For example, Lee *et al.* reconstructed parts of the human heart using collagen-based 3D bioprinting technology.^13^ Sun *et al.* constructed a hepatocellular carcinoma model using 3D bioprinting technology for drug screening.^14^ Compared with traditional tumor models, 3D bioprinted models can simulate tumor heterogeneity by controlling different structures, cell components, and extracellular matrix components, making them more controllable and reproducible. In addition, the gene expression and drug sensitivity of tumor cells in 3D bioprinted models are closer to the real state of patients.^15^

In tumor progression, in addition to tumor cells, the extracellular matrix also plays a key role. It can provide mechanical support for tumor tissues, regulate the microenvironment, and serve as a source of signaling molecules.^16^ Given the central role of ECM in regulating tumor behavior, optimizing biomimetic matrix composition may further improve the physiological relevance and predictive capability of 3D bioprinted CRC models. To improve 3D bioprinted CRC organoid from the perspective of extracellular matrix, we mixed gelatin methacrylate (GelMA) with laminin/entactin complex (LEC) as the extracellular matrix of the 3D bioprinted model. LEC is mainly derived from the Engelbreth-Holm-Swarm (EHS) sarcoma matrix and is a standard medium matrix for organoid construction,^17^ which can provide a suitable matrix environment for organoid culture. The 3D bioprinted CRC organoids constructed using this composite matrix can accurately simulate the tumor microenvironment characteristics and biological behaviors *in vivo*. Furthermore, the reliability of this model in predicting clinical chemotherapy efficacy has been verified through drug sensitivity tests. By obtaining tumor tissue from patients before treatment, this model can reflect the differences in tumor responses to chemotherapeutic drugs among different patients, providing a reference for the formulation of clinical personalized treatment plans. This study aims to provide a new theoretical basis for the precision of CRC treatment from the unique perspective of extracellular matrix and achieve the optimization of CRC treatment.

## II. RESULTS

### A. Effects of different biomaterials on tumor cell biocompatibility, proliferation, and invasion

**FIG. 1.**
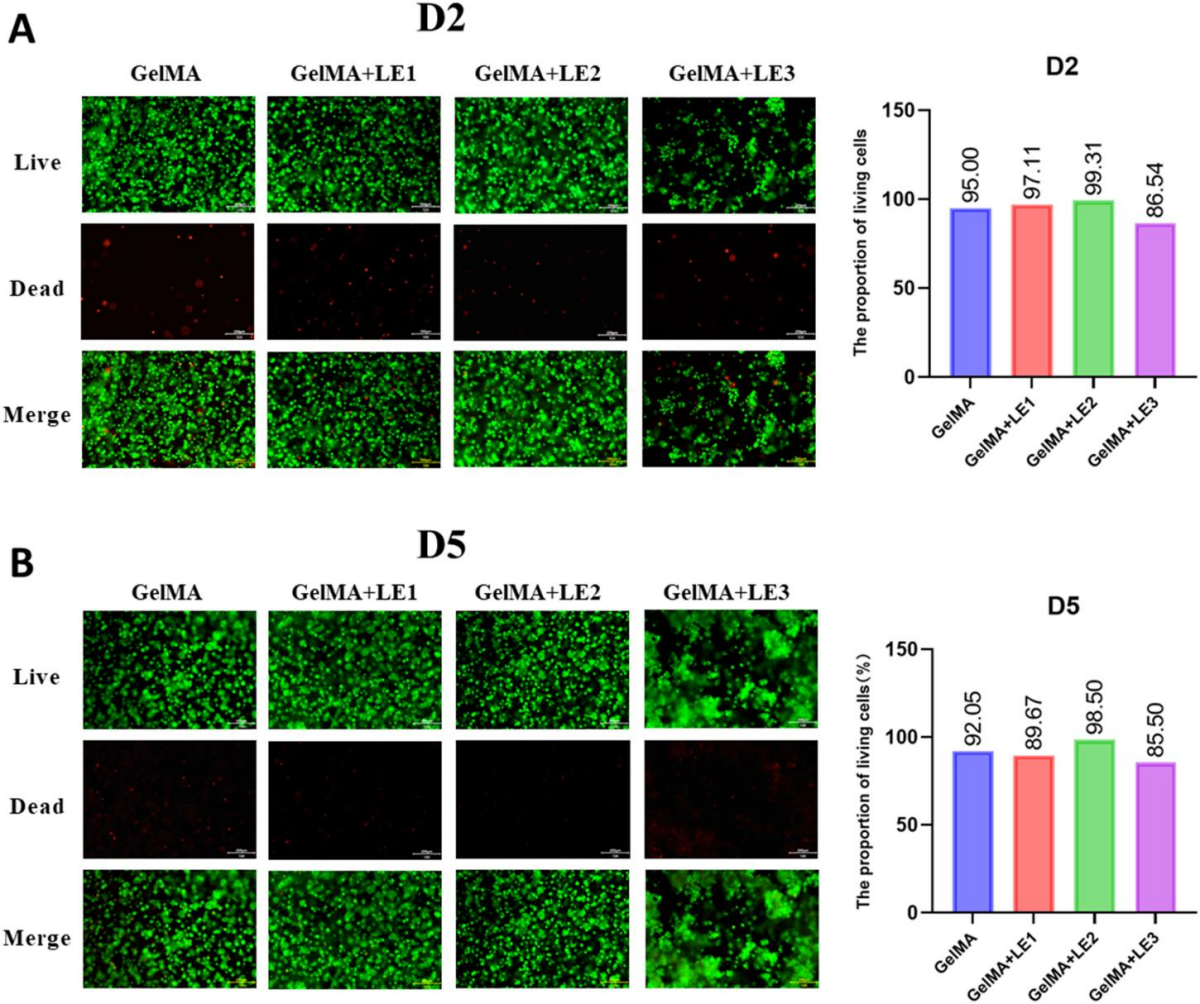
Biocompatibility of different 3D bioprinted materials with the human colorectal cancer cell line SW620. (a) Calcein-AM/propidium iodide staining and quantitative analysis of viable cell ratio of SW620 cells 2 days after 3D bioprinting. (b) Calcein-AM/propidium iodide staining and quantitative analysis of viable cell ratio of SW620 cells 5 days after 3D bioprinting.

First, the biocompatibility of different LEC concentrations with SW620 cells was evaluated by calcein-AM (green for live cells) and propidium iodide (red for dead cells) staining. As shown in Fig. 1, all biomaterials exhibited good biocompatibility on days 2 and 5 of culture. The cell survival rate in the GelMA + LE3 group decreased significantly, while the GelMA + LE2 group maintained the highest cell survival rate. This indicates that the LEC concentration of 1.0 *μ*g/ml (LE2) optimized the biocompatibility of the material. It provides an ideal ratio for subsequent experiments and supports its applicability in tumor model construction.

**FIG. 2.**
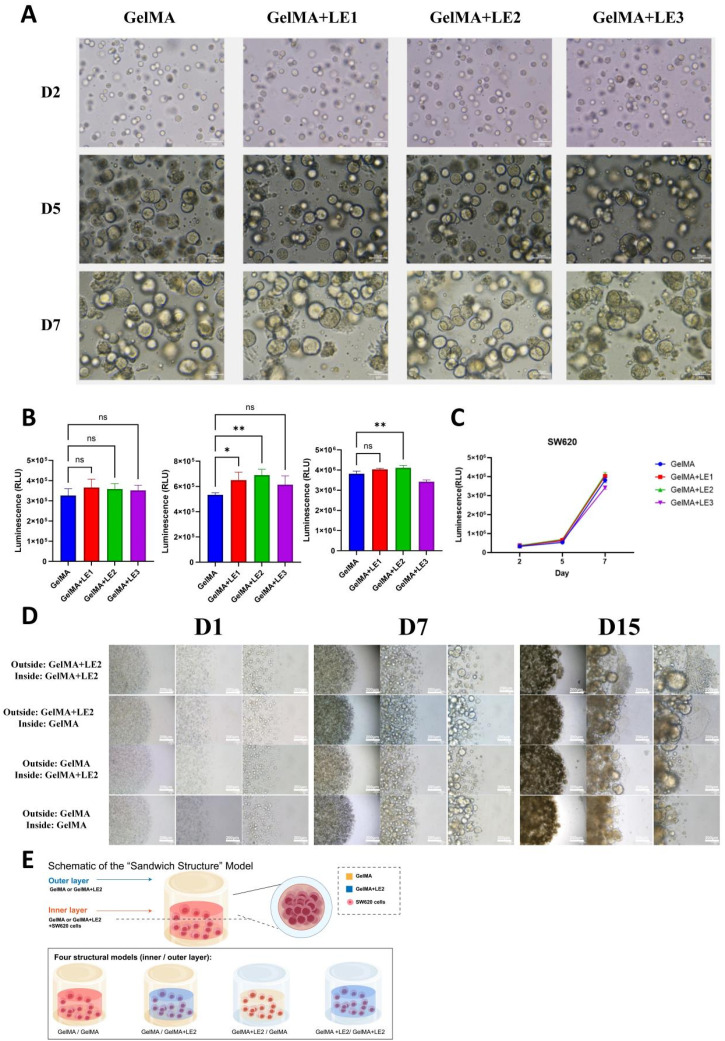
Effects of different 3D bioprinted materials on the proliferation ability of the human colorectal cancer cell line SW620 and primary colorectal cancer cells. (a) Bright-field images of SW620 cells 2, 5, and 7 days after 3D bioprinting. (b) and (c) Relative fluorescence intensity of cells in each group detected by the CellTiter-Glo^®^ Luminescent Cell Viability Assay Kit 2, 5, and 7 days after 3D bioprinting (n = 4). (d) Four structural models with inner and outer layers of GelMA or GelMA + LE2 were constructed. SW620 cell suspension was injected into the inner layer for culture. Bright-field images of cell invasion were taken by confocal microscopy on days 1, 7, and 15. Scale bar = 200 *μ*m. (e) A schematic diagram of the “sandwich structure” model. ^ns^P > 0.05, *P < 0.05, **P < 0.01.

Subsequently, cell proliferation was quantified by bright-field observation and CellTiter-Glo^®^ detection. The results showed that SW620 cells in the GelMA + LE2 group had significant differences from day 5 onward. The fluorescence intensity was significantly higher than that in the control group [Figs. 2(a)–2(c)], indicating that the LEC concentration promotes tumor cell proliferation. To further confirm the advantage of LE2 in promoting tumor cell growth, a “sandwich structure” model with inner and outer layers of GelMA or GelMA + LE2 was constructed [Fig. 2(e)]. The invasion of SW620 cells was observed by confocal microscopy. The results showed that on day 15, the deepest cell invasion was observed when both the inner and outer materials were GelMA + LE2 [Fig. 2(d)]. This indicates that LE2 significantly improves the characteristics of the extracellular matrix. It provides a biomimetic microenvironment for tumor cells, which can most effectively support and promote tumor cell proliferation and invasion. To establish a standardized 3D culture model, a circular sandwich structure consisting of inner and outer layers of GelMA or GelMA + LE2 was designed and used throughout the subsequent experiments [Fig. 2(e)]. The GelMA + LE2 material ratio was selected for subsequent characterization and mechanism research.

### B. Characterization of material properties

**FIG. 3.**
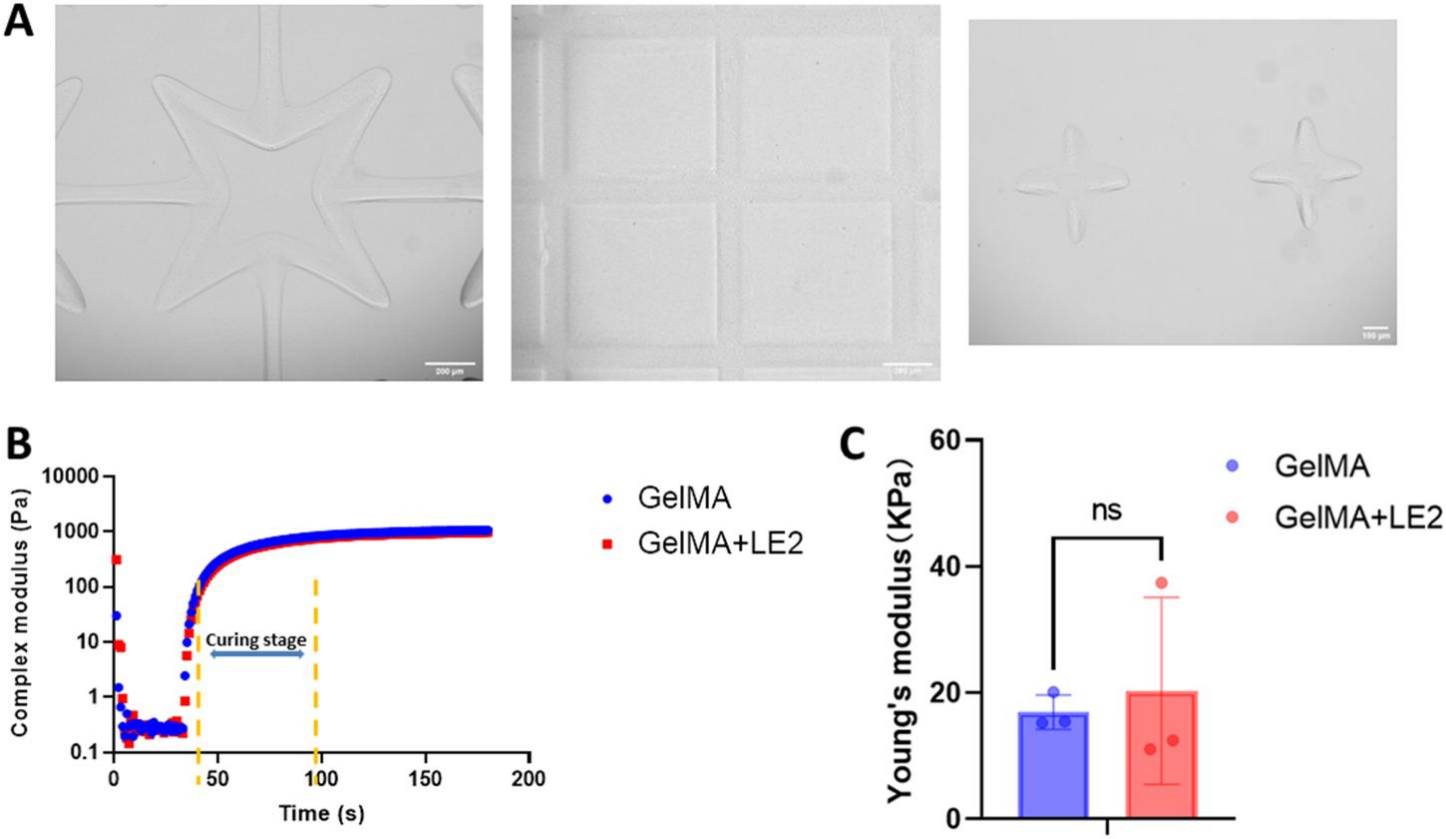
Characterization of the 3D bioprinted material GelMA + LE2. (a) Printing of GelMA + LE2 in molds with different planar patterns. (b) Changes in rheological properties of GelMA and GelMA + LE2 before and after ultraviolet cross-linking detected by a rheometer. (c) Young's modulus of printed models of GelMA and GelMA + LE2 detected by a MicroSquisher (n = 3). ^ns^P > 0.05.

After determining the material ratio of GelMA + LE2, 3D printing was first performed using molds with different planar patterns. As shown in Fig. 3(a), GelMA + LE2 exhibited good planar printability in molds of three patterns. This is advantageous for simulating the structural characteristics of the tumor microenvironment. Subsequently, the rheological properties and Young's modulus of the GelMA + LE2 printed model were characterized. The results showed that after ultraviolet cross-linking, the composite modulus of the material was significantly improved, confirming the enhancement of its mechanical stability [Fig. 3(b)]. However, there was no significant difference between GelMA and GelMA + LE2. Similarly, there was no statistically significant difference detected in their Young's modulus [Fig. 3(c)]. This indicates that LE2 does not change the rheological properties and hardness of the model. Therefore, we speculate that the advantage of LE2 in promoting tumor cell growth is not caused by mechanical changes but by material regulation.

### C. Detection of tumor markers and signaling pathways in 3D bioprinted models

**FIG. 4.**
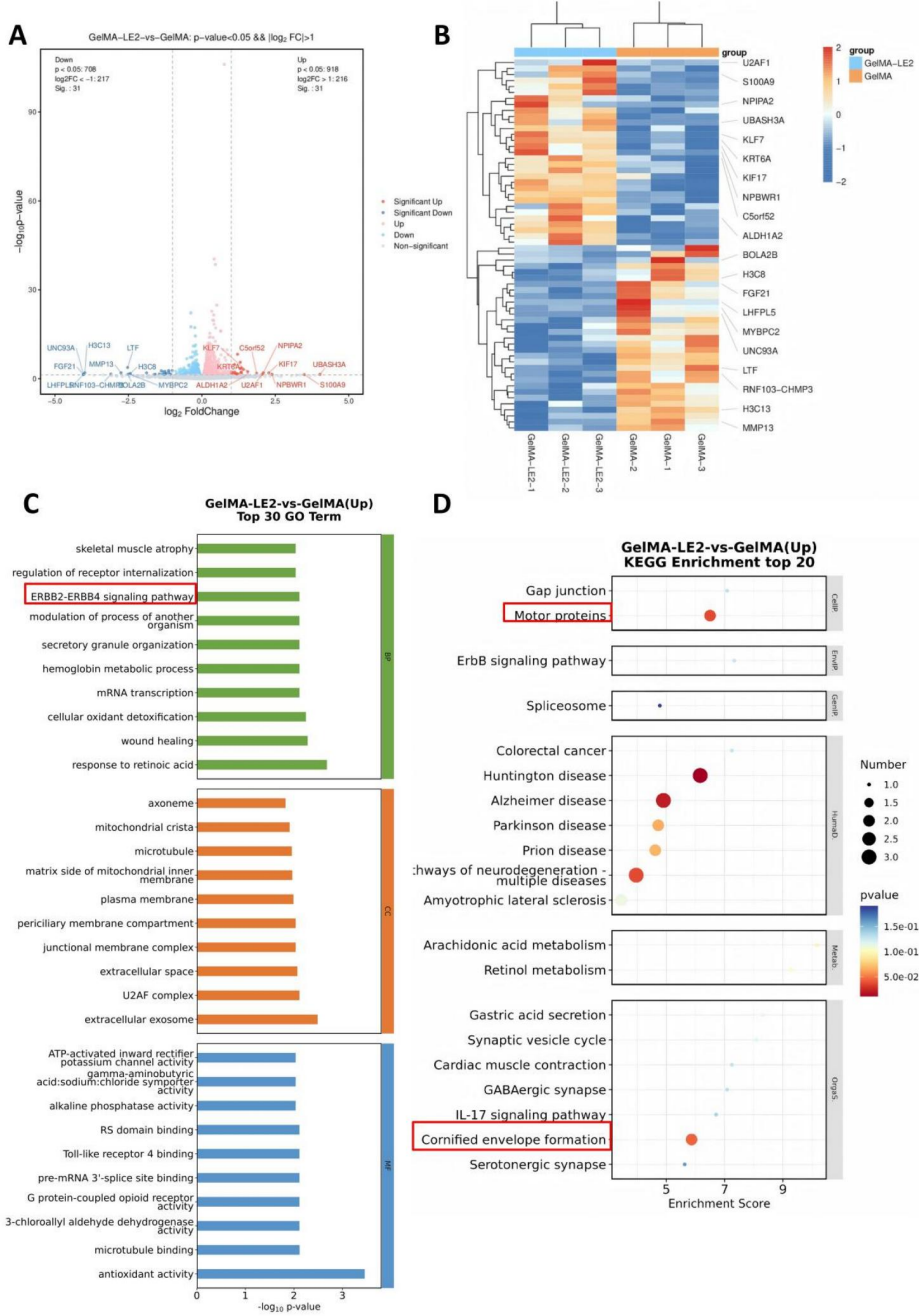
RNA-seq analysis and GO enrichment analysis of differentially expressed genes in SW620 cells between GelMA and GelMA + LE2 models. (a) Volcano plot comparison of transcriptome profiles of cells in the GelMA group and the GelMA + LE2 group. The abscissa represents the log  2-transformed fold change, and the ordinate shows the log-transformed p value corrected by multiple tests. (b) Heatmap of differentially expressed genes in cells between the GelMA group and the GelMA + LE2 group. The color scale indicates the fold change. (c) GO enrichment analysis was performed on the differentially upregulated genes in the GelMA and GelMA + LE2 groups. (d) A KEGG enrichment analysis was performed on the differentially upregulated genes in the GelMA and GelMA + LE2 groups.

To clarify the mechanism of LE2 in regulating tumor cell growth, total RNA was extracted from cells in models printed with GelMA and GelMA + LE2 for RNA-seq. Transcriptome analysis showed significant transcriptome differences between the two groups of samples. PCA (principal component analysis) results separated the GelMA + LE2 group from the control group on the first principal component (49.58% variance) [supplementary material Fig. 1(a)]. Differentially expressed genes (DEGs) were screened using DESeq2 (p < 0.05 and |log_2_FC| > 1) [Figs. 4(a) and 4(b)]. Overall GO enrichment analysis of differentially expressed genes revealed that these genes are primarily associated with extracellular matrix remodeling, inflammatory and immune responses, and growth factor signaling [supplementary material Fig. 1(b)]. Among these, the enrichment of the “collagen catabolic process” suggests that LE2 may promote tumor cell migration and invasion by regulating collagen degradation and extracellular matrix remodeling; the enrichment of “positive regulation of NF-κB transcription factor activity” and “positive regulation of innate immune response” indicates that inflammation-related signaling pathways are activated, which is closely associated with tumor progression and the remodeling of the tumor microenvironment. Furthermore, the enrichment of “fibroblast growth factor receptor binding” and “growth factor activity” suggests that LE2 may enhance growth factor-mediated pro-proliferative and pro-invasive signaling, thereby promoting tumor development.

GO (Gene Ontology) enrichment analysis of the differentially expressed genes revealed that these genes were primarily enriched in pathways associated with biological processes, such as the ERBB signaling pathway. It is an important oncogenic signaling pathway. Its abnormal activation is closely related to almost all cancer characteristics, such as cell proliferation, migration, invasion, and anti-apoptosis [Fig. 4(c)]. Furthermore, KEGG (Kyoto Encyclopedia of Genes and Genomes) analysis revealed that the upregulated genes were significantly enriched in the motor protein and keratinization pathways [Fig. 4(d)]. The enrichment in the motor protein pathway suggests enhanced cytoskeletal dynamics and intracellular transport processes closely associated with cell motility and tumor invasion. The enrichment in the keratinization pathway suggests alterations in the organization of structural proteins and processes related to extracellular barriers, which may reflect changes in extracellular matrix organization and cell adhesion. Taken together, these findings suggest that LE2-induced transcriptomic changes may promote tumor progression by facilitating cytoskeletal remodeling and the reorganization of extracellular structures.

Additionally, a GO enrichment analysis of downregulated differentially expressed genes revealed that these genes were mainly enriched in biological processes like lipopolysaccharide-mediated signaling pathways and collagen degradation when comparing the GelMA + LE2 group with the GelMA group. In particular, the downregulation of lipopolysaccharide-mediated signaling pathways indicates attenuated inflammatory and immune-related responses, which may help tumors evade immune surveillance, while the suppression of collagen catabolism implies decreased collagen degradation, which may encourage extracellular matrix remodeling and create a microenvironment more favorable to tumor cell adhesion, growth, and invasion [supplementary material Fig. 2(a)]. KEGG analysis also showed that downregulated genes were markedly enriched in the pathways for neutrophil extracellular trap (NET) formation and systemic lupus erythematosus [supplementary material Fig. 2(b)], indicating changes in biological processes associated with inflammation and immunity. The burn wound healing pathway was significantly enriched according to the WikiPathways analysis [supplementary material Fig. 2(c)]; on the other hand, pathways linked to actin cytoskeleton regulation and FBXL10-mediated enhancement of the MAPK/ERK (Mitogen-Activated Protein Kinase/Extracellular Signal-Regulated Kinase) signaling pathway in non-Hodgkin lymphoma showed a downregulated trend [supplementary material Fig. 2(d)], indicating possible changes in cytoskeletal structure, cell adhesion, and migration functions. Reactome analysis also showed significant enrichment of the RMTs (arginine methyltransferases) methylate histone arginines pathway [supplementary material Fig. 2(f)] and enrichment of the Neuronal System and Transmission across Chemical Synapses pathways [supplementary material Fig. 2(e)], indicating that LE2 may also affect epigenetic regulatory processes and intercellular communication. Together, these results suggest that LE2-induced transcriptomic changes may involve coordinated regulation of extracellular matrix remodeling, immune and inflammatory responses, cytoskeletal dynamics, intercellular signaling, and epigenetic regulation, all of which may contribute to the invasion, proliferation, and remodeling of the tumor microenvironment.

Furthermore, KEGG enrichment analysis of all differentially expressed genes (DEGs) revealed that DEGs were significantly enriched in inflammation- and immunity-related pathways, such as necroptosis, the IL-17 signaling pathway, and neutrophil extracellular trap (NET) formation, suggesting that LE2 may participate in the remodeling of the tumor microenvironment by regulating inflammatory responses and programmed cell death [supplementary material Fig. 1(c)]. WikiPathways analysis further revealed significant enrichment in pathways such as FBXL10-mediated enhancement of MAPK/ERK signaling, miRNA targets in the ECM and membrane receptors, and the senescence-associated secretory phenotype (SASP), suggesting that LE2 may promote tumor progression by activating MAPK/ERK signaling, regulating ECM-related miRNA networks, and modulating the inflammatory secretory phenotype [supplementary material Fig. 1(d)]. Additionally, Reactome analysis revealed that differentially expressed genes were significantly enriched in pathways including Metal sequestration by antimicrobial proteins and RMTs methylate histone arginines. These findings suggest that LE2 may influence tumor progression through coordinated regulation of antimicrobial and immune-associated responses as well as epigenetic modification [supplementary material Fig. 1(e)]. Multi-database pathway enrichment analysis indicates that LE2-induced transcriptional changes primarily involve extracellular matrix remodeling, the regulation of inflammatory and immune responses, dynamic changes in the cytoskeleton, and the activation of various classical oncogenic signaling pathways, which collectively promote tumor cell proliferation, migration, invasion, and tumor microenvironment remodeling.

### D. Effects of composite biomaterials on the proliferation of primary CRC cells

**FIG. 5.**
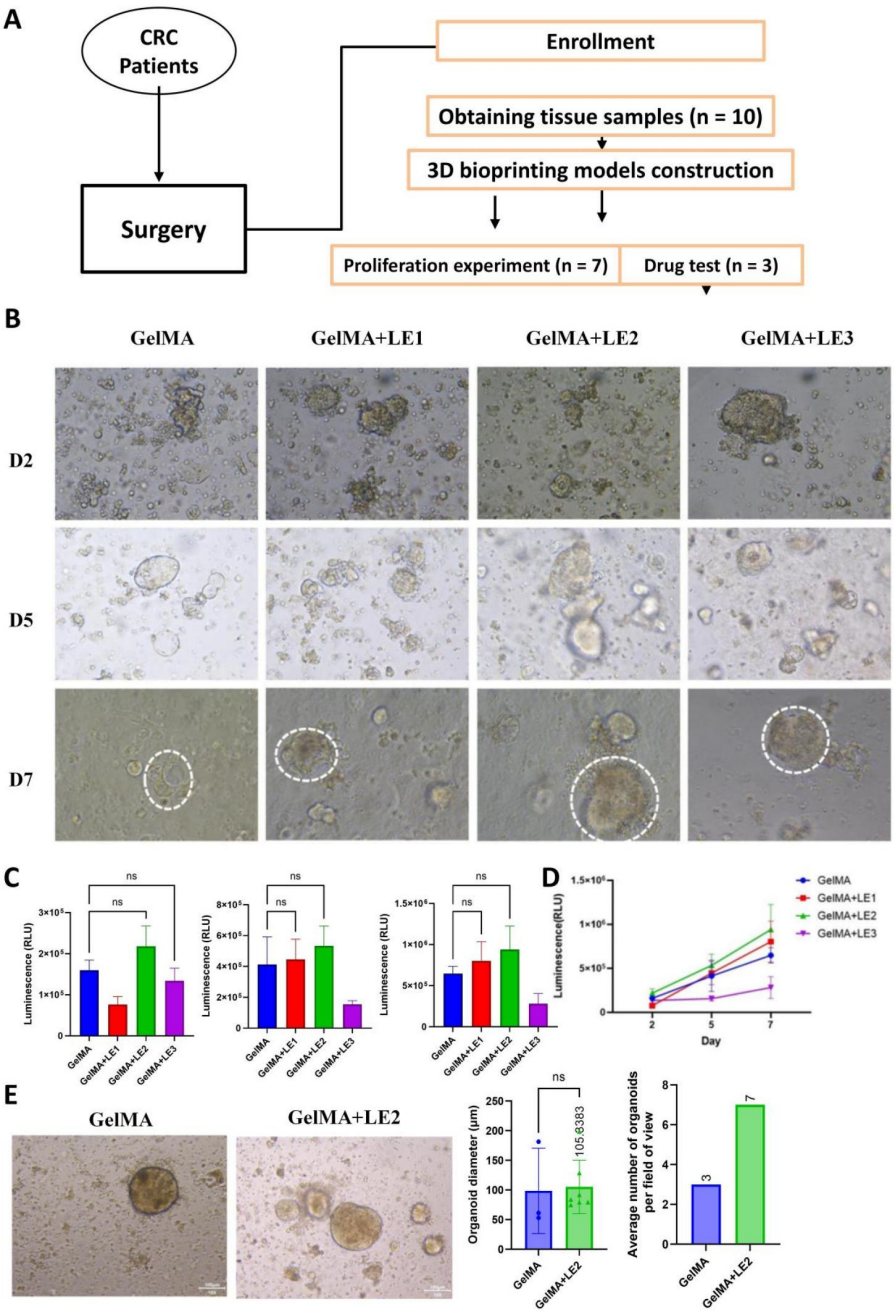
Effects of different 3D bioprinted materials on the proliferation ability of primary colorectal cancer cells. (a) Research process of colorectal cancer. A total of 10 patient samples undergoing CRC surgery were enrolled, of which 7 were used for proliferation experiments and 3 for drug testing. (b) Bright-field images of primary colorectal cancer cells 2, 5, and 7 days after 3D bioprinting. (c) and (d) Relative fluorescence intensity of cells in each group detected by the CellTiter-Glo^®^ Luminescent Cell Viability Assay Kit 2, 5, and 7 days after 3D bioprinting (n = 4). (e) Representative images of the growth patterns of colorectal cancer organoids produced by 3D printing, along with quantitative analyses of the number and diameter of the organoids. ^ns^P > 0.05.

To further verify the promoting effect of composite biomaterials on tumor cell proliferation, primary CRC cells were extracted from patient-derived colorectal cancer tissues for proliferation experiments [Fig. 5(a)]. The results showed that there was no significant difference in the fluorescence intensity of cells in each group on day 7. However, a significant enhancement in cell proliferation was observed in the GelMA + LE2 group [Figs. 5(b)–5(d)]. This is consistent with the results of SW620 cells, proving that the LE2 material has a universal proliferation-promoting effect on patient-derived cells. In addition, to further evaluate the effect of composite materials on the three-dimensional culture of patient-derived CRC cells, we observed organoid formation in different groups [Fig. 5(e)]. The results showed that, compared with the GelMA group, the GelMA + LE2 group formed a greater number of organoids (approximately 7 vs 3) and exhibited a more compact growth pattern. Although statistical analysis was performed on organoid diameter, due to significant individual heterogeneity and morphological variations among patient-derived organoids, the changes in diameter did not reveal clear intergroup differences. Therefore, the number of organoids formed and observations of their overall morphology better reflect the beneficial effect of the LE2 material on the 3D culture system. These results further support that LE2 can optimize the 3D culture microenvironment and promote the formation and maintenance of patient-derived CRC cell organoids.

### E. Chemotherapeutic drug testing of CRC organoids based on GelMA + LE2 3D bioprinting

**FIG. 6.**
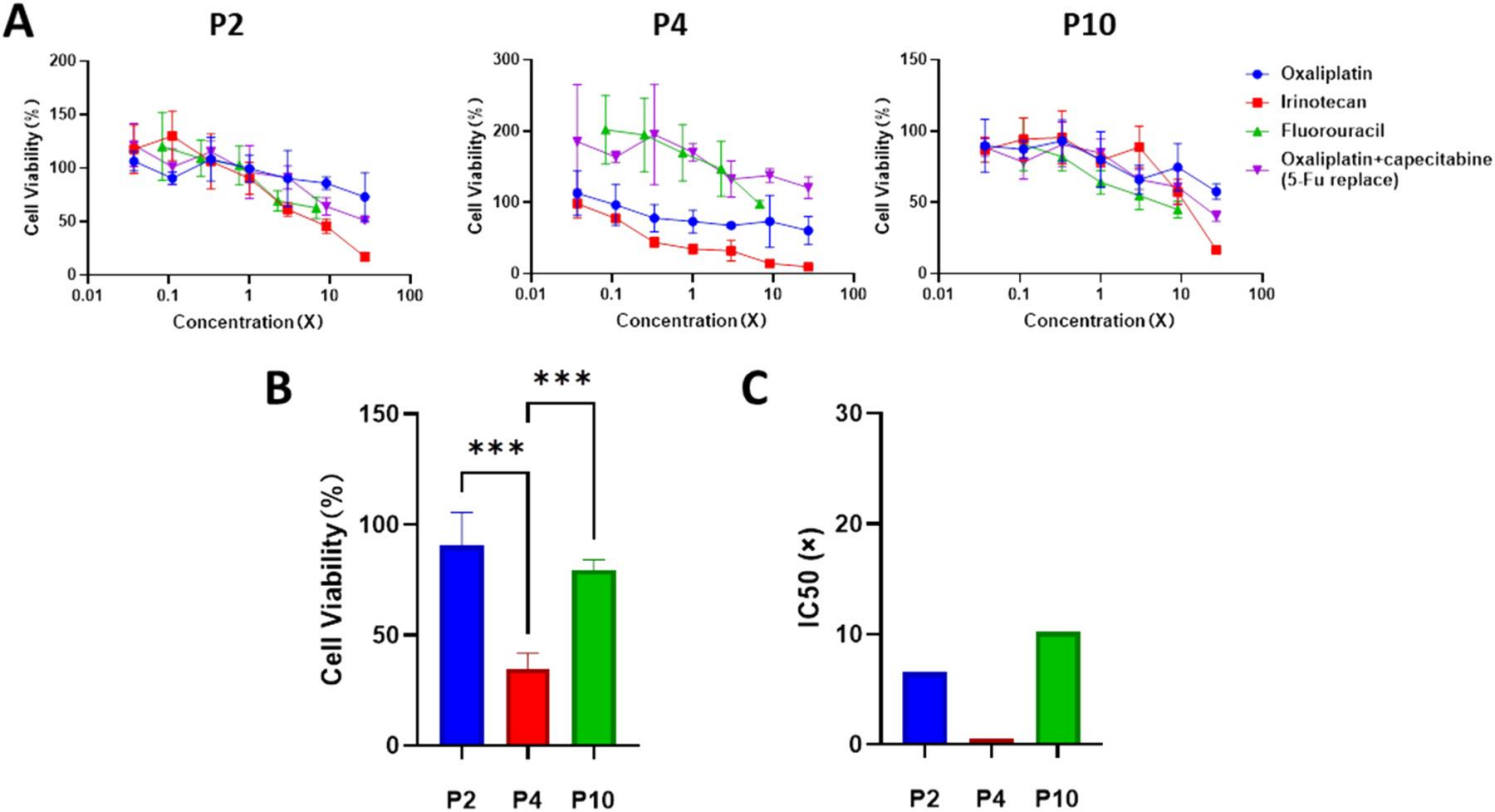
Clinical drug evaluation of 3D printed CRC organoids. (a) Efficacy evaluation of 3 clinical samples, including 2 cases of T3N0M0 (P2 and P10 samples) and 1 case of T2N0M0 (P4 sample) (n = 3). (b) Efficacy evaluation of the three samples at the same irinotecan concentration (1 × concentration) (n = 3). (c) Differences in irinotecan IC_50_ among the three samples. ***P < 0.001.

To evaluate the clinical transformation value of 3D printed primary CRC organoids, four first-line chemotherapy regimens for CRC were used for drug sensitivity testing. As shown in Fig. 6(a), irinotecan was the most effective and widely used single agent among all tested drugs. It was the only treatment regimen that showed sensitivity in all three organoid lines. Moreover, the sensitivity was significantly associated with the tumor T stage. The P4 organoid line (stage T2N0M0) showed significantly higher sensitivity to irinotecan (IC_50_ = 3.02 *μ*M). In contrast, the P2 and P10 organoid lines (both stage T3N0M0) showed moderate sensitivity (IC_50_ = 37.83/89.96 *μ*M) [Figs. 6(b) and 6(c)]. On the contrary, no sensitivity to oxaliplatin was detected in any of the three organoid lines. Its IC_50_ value exceeded the detection range, suggesting that this drug may not be suitable for such patients. In addition, the efficacy of fluorouracil and the oxaliplatin + capecitabine (5-Fu substitution) combination regimen was significantly limited and only applicable to specific organoids. Fluorouracil showed moderate sensitivity only in the P10 organoid line (IC_50_ = 89.96 *μ*M) but was not sensitive in the P2 and P4 organoids (IC_50_ exceeded the detection range). The oxaliplatin + capecitabine (5-Fu substitution) combination regimen showed moderate sensitivity in P2 (oxaliplatin 118.49 *μ*M, 5-Fu 128.53 *μ*M) and P10 (oxaliplatin 87.49 *μ*M, 5-Fu 94.90 *μ*M) organoids. However, it was not sensitive in the P4 organoid and did not show synergistic efficacy superior to irinotecan monotherapy.

Clinical follow-up results indicated that all three patients received clinical chemotherapy regimens formulated based on the drug sensitivity testing results of the 3D bioprinted organoids, and all achieved Complete Response (CR) after treatment. This confirms a good correspondence between the drug sensitivity test results of the model and clinical treatment responses, further verifying the reliability of the model in predicting clinical chemotherapy efficacy.

## III. DISCUSSION

The biomimetic nature and clinical transformation ability of preclinical CRC models are the core bottlenecks restricting precision treatment research. Traditional 2D cell models cannot reproduce tumor cell heterogeneity and invasive phenotypes due to the lack of extracellular matrix (ECM) regulation and single culture requirements.^18^ Although patient-derived xenograft (PDX) models can simulate the *in vivo* microenvironment, they have defects such as long culture cycles (needing weeks to months), significant species differences, and low success rates.^19^ Patient-derived organoid (PDTO) models have overcome some limitations but still face problems such as the lack of standardized culture systems and difficulty in precisely regulating ECM components.^20^ Therefore, there remains a need for a CRC model that combines biomimetic tumor microenvironments, structural reproducibility, and clinically applicable drug screening capability. Based on 3D bioprinting technology, this study constructed a CRC organoid model with biomimetic ECM using gelatin methacrylate (GelMA) and laminin/entactin complex (LEC). The advantages and limitations of the GelMA + LE2 model relative to existing preclinical CRC models are summarized in supplementary material Table 2. It achieved targeted breakthroughs in the material design, cell behavior regulation, and clinical transformation.

From the perspective of material engineering, this study selected GelMA as the base material. It has both photocurable properties and good biocompatibility, which can precisely control the mechanical stability of the printed structure through ultraviolet cross-linking. As a natural ECM component derived from the EHS sarcoma matrix, LEC is one of the gold standard matrices for organoid culture.^17^ The core advantage of their combination is to achieve a balance between structural controllability and biomimetic nature. Crucially, the GelMA/LEC system replicates a number of important ECM characteristics linked to the development of colorectal cancer, despite not being able to fully recreate the biochemical complexity of the native colorectal ECM.^21,22^ Collagen, glycoproteins, proteoglycans, glycosaminoglycans (GAGs), and basement membrane proteins makeup the highly organized network that makes up the healthy colorectal ECM.^23^ Extensive ECM remodeling, which is marked by increased fibrillar collagen deposition and cross-linking, increasing GAG levels, increased production of matrix glycoproteins, and progressive matrix stiffness, takes place during the evolution of colorectal tumors.^24,25^ In this model, LECs supply elements linked to the basement membrane, such as laminin, type IV collagen, and heparan sulfate proteoglycans, whereas GelMA supplies a collagen-rich scaffold that partially replicates the increased collagen accumulation seen in the tumor stroma. As a result, the GelMA/LEC composite matrix preserves the simplicity and repeatability needed for 3D bioprinting applications while capturing a number of important structural characteristics of the colorectal tumor microenvironment.

The results showed that when the LEC concentration was 1.0 *μ*g/ml (GelMA + LE2 group), the survival rates of SW620 cells and primary CRC cells were the highest. Moreover, planar printing experiments confirmed that this material can stably reproduce preset patterns, laying a foundation for simulating the spatial structural heterogeneity of the tumor microenvironment. It is worth noting that rheological and elastic tests showed no significant difference in mechanical properties between GelMA + LE2 and pure GelMA. This suggests that the promoting effect of LE2 on tumor cell proliferation and invasion is not caused by changes in the mechanical microenvironment but by regulating cell signaling pathways through ECM components. This finding excludes the interference of mechanical factors on the experimental results and clarifies the biological regulatory value of LE2.

To further elucidate the biological basis underlying the enhanced tumor phenotypes observed in the GelMA + LE2 model, transcriptome sequencing and pathway enrichment analysis using multiple databases jointly revealed the mechanism of LE2. First, the ErbB signaling pathway [including the EGFR/HER (Human Epidermal Growth Factor Receptor) family] was significantly upregulated. It is one of the most common oncogenic pathways in CRC.^26^ Its abnormal activation can promote tumor cell proliferation, anti-apoptosis, and epithelial–mesenchymal transition (EMT) through downstream PI3K-Akt signals.^6,27^ Second, the Wnt signaling pathway was activated. This pathway plays a core role in maintaining the stemness of cancer stem cells and regulating cell migration. It is closely related to the invasion depth and lymph node metastasis of CRC.^7,28^ Third, gap junction-mediated intercellular communication was enriched. This process can enhance signal transmission and migration ability between tumor cells.^29^ It provides a molecular explanation for the phenomenon of the deepest cell invasion observed when both the inner and outer layers involve GelMA + LE2 in the model. Collectively, these findings suggest that the GelMA + LE2 matrix may influence CRC cell proliferation and invasion through multiple signaling pathways associated with tumor progression. Although the current data do not establish direct causal relationships between ECM composition, pathway activation, and tumor phenotypes, they provide preliminary mechanistic insights and a basis for future functional validation studies.

Beyond mechanistic exploration, evaluating the potential translational applicability of the model is equally important. In this proof-of-concept study, we preliminarily assessed whether the 3D bioprinted CRC organoid model could reflect interpatient differences in chemotherapy response. Tests of four first-line CRC chemotherapy regimens showed that irinotecan was the only drug that showed sensitivity in all three primary organoid lines. Moreover, the sensitivity was significantly associated with the tumor T stage. The P4 sample of stage T2 (invasion into the muscularis propria) had the highest sensitivity to irinotecan. In contrast, the P2 and P10 samples of stage T3 (invasion through the muscularis propria to the subserosa) had significantly reduced sensitivity. This is consistent with the clinical observation that the deeper the tumor invasion, the lower the chemotherapy sensitivity may be.^30^ More importantly, a good correspondence was formed between the drug sensitivity test results of the model and the clinical treatment responses of the corresponding patients: all patients achieved Complete Response (CR) after treatment based on the sensitive regimens predicted by the model. Although based on a limited number of patient-derived organoids, the observed consistency between *in vitro* drug sensitivity and patient treatment responses suggests the potential utility of the model for individualized chemotherapy screening. However, these findings should require validation in larger patient cohorts.

These preliminary findings indicate that the current model may have translational potential in individualized CRC treatment. Based on this correlation, this study further drew important insights for drug screening in colorectal cancer. For one thing, 3D bioprinted organoids can serve as a pre-experimental tool for clinical chemotherapy regimen selection. They may enable rapid *in vitro* evaluation of potential drug sensitivity before treatment initiation, avoid the blindness of trial-and-error medication in traditional therapy, and provide direct references for the formulation of individualized treatment regimens. For another, the observed association between irinotecan sensitivity and tumor T stage further suggests that tumor invasion depth may influence chemotherapy response, although this observation requires validation in larger studies. For T2-stage tumors with localized invasion, irinotecan monotherapy may be sufficient to achieve desirable efficacy. For T3-stage tumors with deep invasion, combination with other moderately sensitive drugs based on irinotecan or adjusted administration dose can be considered to improve therapeutic efficacy. Additionally, the model confirmed that oxaliplatin showed no sensitivity in the patient cohort of this study. This indicates that such patients can avoid the administration of this drug, which not only reduces the waste of medical resources caused by ineffective treatment but also alleviates the harm of adverse drug reactions to patients. Notably, combination regimens did not show superior synergistic efficacy to irinotecan monotherapy. This suggests that combination therapy is not necessarily more optimal in the individualized treatment of colorectal cancer. Therefore, individualized drug screening strategies based on patient-derived models may help optimize treatment selection and reduce unnecessary treatment exposure.

Despite these encouraging findings, several limitations should be acknowledged when interpreting the current results. First, the sample size is small (10 clinical patients, 3 primary organoids used for drug testing). Future studies need to expand the sample size to verify the universality of the model. Second, the model does not include immune cells (such as T cells and macrophages) and vascular endothelial cells. It cannot simulate the immunosuppressive microenvironment of CRC and tumor angiogenesis. These two factors are key variables affecting clinical chemotherapy efficacy.^31,32^ Furthermore, anatomical site-specific heterogeneity may also lead to differences in CRC tumor behavior and drug response.^33^ Previous studies have shown that proximal CRC and distal CRC exhibit significant differences in terms of immune infiltration, mucinous characteristics, and matrix/ECM remodeling,^34,35^ and these differences may further influence cellular behavior and drug sensitivity in 3D-bioprinted CRC models. Although this study did not perform a stratified analysis by anatomical location, this factor may be an important source of model heterogeneity and requires further validation in larger cohorts. In addition, drug testing only evaluated short-term efficacy. It lacks long-term drug resistance monitoring and cannot fully reproduce the phenomenon of initial sensitivity followed by drug resistance in clinical practice. Future studies can further improve the clinical prediction accuracy of the model by constructing co-culture models of tumor cells, immune cells, and vascular endothelial cells and extending the observation period.

## IV. CONCLUSION

This study successfully constructed a 3D bioprinted CRC organoid model based on GelMA + LE2. The model has good biocompatibility, printability, and biomimetic microenvironmental characteristics. It can effectively promote tumor cell proliferation and invasion by activating the ErbB/Wnt signaling pathway and gap junction communication. Drug tests confirmed that the model can distinguish the chemotherapy sensitivity of CRC with different T stages. The results are correlated with clinical pathological characteristics, providing a reliable platform for personalized drug screening. In summary, the 3D bioprinted model constructed in this study not only overcomes the limitations of traditional CRC models but also establishes a research framework of “ECM biomimetic design—molecular mechanism—clinical transformation.” It provides a new technical scheme and theoretical basis for preclinical research on CRC precision medicine.

## V. METHODS

### A. Participants and specimens

All human tissue collection and experiments have been approved by the Institutional Review Board and the Health Insurance Portability and Accountability Act, and are in compliance with the relevant requirements of the Declaration of Helsinki. All enrolled patients or their legal representatives signed written informed consent forms prior to specimen collection.

This study enrolled 10 CRC patients who underwent surgical treatment at our hospital in 2025. All patients were diagnosed with CRC by postoperative pathological examination. They had not received anti-tumor treatments such as radiotherapy or chemotherapy before surgery, had no history of other malignant tumors, and had complete clinical medical records. The baseline characteristics of the patients are shown in Table I.

**TABLE I.** Clinical characteristics of patients with colorectal cancer.

	Sex	Age (years)	Histopathology	Primary site	TNM stage	Tumor size
P1	Female	75	Adenocarcinoma	Rectum	T2N0M0	2 × 1.3 × 0.4 cm^3^
P2	Male	74	Adenocarcinoma	Ascending colon	T3N0M0	5 × 3.5 × 0.8 cm^3^
P3	Male	75	Adenocarcinoma	Colon hepatic flexure, sigmoid colon	T3N1M0	Colon hepatic flexure: 4 × 3.5 × 1 cm^3^; Sigmoid colon: 3 × 2.5 × 2 cm^3^
P4	Female	60	Adenocarcinoma	Right colon	T2N0M0	4 × 3.5 × 1 cm^3^
P5	Male	60	Adenocarcinoma	Sigmoid colon	T3N0M0	4 × 2 × 1.5 cm^3^
P6	Male	82	Adenocarcinoma	Sigmoid colon	T2N0M0	3 × 2 × 1 cm^3^
P7	Female	75	Adenocarcinoma	Rectum	T4N0M0	4 × 3 × 1.5 cm^3^
P8	Male	68	Adenocarcinoma	Rectum	T4N0M0	5.5 × 5.5 × 1.5 cm^3^
P9	Male	65	Adenocarcinoma, partial mucinous adenocarcinoma	Rectum	T4N1M0	4 × 4 × 0.8 cm^3^
P10	Male	84	Adenocarcinoma	Ascending colon	T3N0M0	4.5 × 4 × 1 cm^3^

All specimens were fresh colorectal cancer tissues resected from patients. Surgical methods included laparoscopic radical resection of rectal cancer, laparoscopic radical resection of right colon cancer, and subtotal colectomy (specific surgical plans were formulated individually according to the patient's condition). Immediately after collection, specimens were placed in cold phosphate-buffered saline (PBS) containing 1% penicillin–streptomycin and transported to the laboratory within 2 h for subsequent tumor cell isolation and 3D bioprinted model construction. The specimen collection process strictly followed sterile operating specifications to avoid tissue contamination and necrosis. To account for potential site-specific heterogeneity, colorectal cancer specimens were additionally categorized according to their anatomical origin. Detailed anatomical classification and pathological characteristics are provided in supplementary material Table 1.

### B. Cell isolation and culture

In a biosafety cabinet, tissues used for tumor cell isolation and culture were washed three to five times with cold PBS (Servicebio, G4207-500ML) containing 1% penicillin–streptomycin (Servicebio, G4016-100ML) to remove surface blood stains, necrotic tissues, and bacteria. The tissues were cut into small pieces of 1–3 mm^3^ on ice with a sterile scalpel. Tissue digestion medium (DMEM, Dulbecco's Modified Eagle Medium)/F12 medium supplemented with 1.5 mg/ml collagenase II, 0.15 mg/ml hyaluronidase, and 1% penicillin–streptomycin) was added. The mixture was incubated in a 37 °C incubator for 1–2 h with manual shaking every 15 min. Subsequently, it was filtered through a 100 *μ*m cell strainer to remove undigested tissue debris. The filtered cell suspension was centrifuged at 1200 rpm for 5 min. After cell counting, the cells were resuspended in PBS for subsequent bioprinting. In addition, the human colorectal cancer cell line SW620 (BNCC337664) was cultured in complete DMEM medium (Thermo Fisher, 11965092).

### C. Preparation of 3D bioprinting materials

In this study, gradient concentrations of LEC combined with photocurable GelMA hydrogel were used to prepare 3D bioprinting bioinks with biomimetic ECM properties. The specific preparation process was as follows: 50 *μ*l of 10% GelMA (Aladdin, M752743) was mixed with 5 *μ*l of 2% photoinitiator lithium phenyl-2,4,6-trimethylbenzoylphosphinate (LAP, Aladdin, l157759) and stirred at 37 °C in the dark for 10 min. Then, empty EP tubes were taken to add gradient volumes of PBS/cell suspension (40.8, 41.6, and 28.2 *μ*l). After pre-cooling at 4 °C, corresponding volumes of LEC solution (4.2, 8.4, 16.8 *μ*l, and 12 mg/ml) were added. After balancing at room temperature for 5 min, 55 *μ*l of pre-mixed GelMA + LAP solution was added to each EP tube and thoroughly mixed. GelMA bioinks with LEC concentrations of 0/0.5/1.0/2.0 *μ*g/ml and a cell concentration of 5 × 10^6^ cells/ml were obtained. Consequently, the final composition of the bioinks was 5% (w/v) GelMA for the GelMA group, 5% GelMA supplemented with 0.5 mg/ml LEC for the GelMA + LE1 group, 5% GelMA supplied with 1.0 mg/ml LEC for the GelMA + LE2 group, and 5% GelMA supplemented with 2.0 mg/ml LEC for the GelMA + LE3 group. LEC produced from Engelbreit-Holm-Swarm (EHS) sarcoma contains extracellular matrix components associated with the basement membrane, such as laminin, type IV collagen, and heparan sulfate proteoglycans. GelMA, a gelatin derivative, mainly offers a collagen-like structural framework.

### D. Construction of 3D bioprinted colorectal cancer models

The prepared GelMA bioink was aspirated into a syringe and incubated at 4 °C for 30 min until the ink formed a gel. The syringe was then installed on an Azure photocurable 3D bioprinter (CYBERIAD). The nozzle temperature and chamber temperature were set to 20 and 10 °C, respectively. Printing was performed at an extrusion speed of 1.5 mm^3^/s, with cross-linking under 405 nm ultraviolet light (0.5 W/cm^2^) for 50 s. The entire printing process was completed within 2 h. The printed 3D bioprinted models were collected in 48-well plates on the chamber platform. For biocompatibility and proliferation assays, 2 *μ*l of cell-laden bioink was dispensed into each well of a 96-well plate and photocrosslinked using a 405 nm light source for 25 s. After cross-linking, 100 *μ*l of complete culture medium was added to each well. For invasion assays, 5 *μ*l of cell-laden bioink was printed in the center of each well of a 48-well plate and surrounded by 20 *μ*l of cell-free bioink serving as the invasion matrix. Constructs were photocrosslinked under 405 nm light for 20 s and cultured in 500 *μ*l of complete medium. To ensure consistency between wells and batches, all bioinks were prepared using identical formulations and cell densities, and printing was performed using the same predefined digital pattern and exposure parameters. All constructs within the same experiment were fabricated in a single printing batch under identical environmental conditions.

### E. Evaluation of model mechanical properties

A DHR rheometer was used to evaluate the rheological properties of the 3D bioprinted models. GelMA + LE bioink was injected into the parallel plate fixture of the rheometer to ensure the sample surface was flat and free of bubbles. It was balanced at 37 °C for 5 min. Baseline measurement of initial rheological parameters was completed in the absence of light. Subsequently, ultraviolet light irradiation at 405 nm (0.5 W/cm^2^) was performed to monitor changes in rheological parameters during cross-linking and analyze the sol–gel transition of the material. A MicroSquisher was used to measure the elastic modulus of the 3D bioprinted models. For mechanical testing, acellular constructs were prepared using the same bioink formulation as described earlier, except that the cell suspension was replaced with sterile PBS. A total of 10 *μ*l bioink was deposited into each well and photocrosslinked to form cylindrical hydrogel constructs (diameter 0.6 mm and height 0.6 mm). Mechanical testing was performed on day 1 to evaluate the stability of the model components. During measurement, the model was compressed once through a stainless steel beam and plate by a displacement of 17% of its height within 25 ms, and the height of the model after compression recovery was measured for calculating Young's modulus.

### F. Biocompatibility, proliferation, and invasion

To evaluate the biocompatibility of different LEC concentrations with tumor cells, calcein-AM and propidium iodide staining (Beyotime, C2015S) were performed on days 2 and 5 after printing to distinguish live cells (green) and dead cells (red). For cell proliferation, evaluation was performed using the CellTiter-Glo^®^ Luminescent Cell Viability Assay Kit (Promega, G7570) on days 2, 5, and 7 according to the manufacturer's instructions. The fluorescence intensity was measured by microplate reader (CYBERIAD).

To evaluate the effect of GelMA + LE2 composite material on the invasion ability of colorectal cancer cells, four structural models with inner and outer layers of GelMA or GelMA + LE2 were constructed. SW620 cell suspension was injected into the inner layer for culture. The invasion depth of cells was observed by confocal microscopy on days 1, 7, and 15.

### G. RNA sequencing and pathway analysis

For RNA-seq analysis, total RNA was extracted from cells of the GelMA group and the GelMA + LE2 group after 7 days of 3D printed culture using the TRIzol method. mRNA was enriched via PolyA selection, and RNA-seq was performed on the high-throughput Illumina sequencing platform Illumina NovaSeq 6000. Differentially expressed genes (DEGs) were screened using the DESeq2 software with the thresholds of p < 0.05 and |log_2_FC| > 1. A total of 62 DEGs were identified, including 31 upregulated genes and 31 downregulated genes. After obtaining DEGs, GO enrichment analysis was conducted to characterize their functions. Subsequently, pathway enrichment analysis of DEGs was performed using KEGG, Reactome, and WikiPathways databases, with a focus on pathways related to tumor proliferation and invasion.

### H. Drug testing

Four first-line chemotherapy regimens for CRC were used for drug sensitivity testing, including single agents (irinotecan, oxaliplatin, and fluorouracil) and a combination regimen [oxaliplatin + capecitabine, with 5-fluorouracil (5-Fu) as a substitute]. The three primary organoid lines were derived from patients with pathologically confirmed moderately differentiated adenocarcinoma (all N0M0, no distant metastasis). They included two stage T3 samples (P2 and P10, tumor invasion through the muscularis propria to the subserosa) and one stage T2 sample (P4, tumor invasion into the muscularis propria), allowing for precise comparison of drug response differences across different tumor invasion depths.

### I. Statistical analysis

GraphPad Prism 8.0 was used for statistical analysis. Data were expressed as mean ± standard deviation (SD). Differences among multiple groups were analyzed using one-way analysis of variance (ANOVA). Statistical analysis was performed using data from at least three independent experiments. P < 0.05 was considered statistically significant. Screening of differentially expressed genes was performed using the DESeq2 package (version 1.30.0) in the R environment (version 4.0.3), with P < 0.05 and |log_2_FC| > 1 as the significance thresholds. Gene enrichment analysis was completed using the clusterProfiler package (version 3.18.0), with FDR < 0.05 defined as significant. The correlation between clinical treatment outcomes and drug responses of 3D bioprinted models was evaluated using Pearson correlation coefficient or Spearman rank correlation coefficient. The correlation strength was expressed as the r value, and the 95% confidence interval was calculated.

## SUPPLEMENTARY MATERIAL

See the supplementary material for the following: Supplementary Fig. 1. Transcriptomic analysis of the GelMA and GelMA + LE2 models; supplementary transcriptomic analysis plots comparing gene expression profiles of cells cultured in GelMA and GelMA + LE2 models; Supplementary Fig. 2. Pathway enrichment analysis of differentially expressed genes between the GelMA and GelMA + LE2 groups; Supplementary material Table I. Anatomical site classification and pathological characteristics of colorectal cancer specimens; and Supplementary material Table II. Comparison of different preclinical CRC models.

## Data Availability

The data that support the findings of this study are available from the corresponding author upon reasonable request.
